# Spatial distribution of malignant transformation in patients with low-grade glioma

**DOI:** 10.1007/s11060-020-03391-1

**Published:** 2020-01-09

**Authors:** Asgeir S. Jakola, David Bouget, Ingerid Reinertsen, Anne J. Skjulsvik, Lisa Millgård Sagberg, Hans Kristian Bø, Sasha Gulati, Kristin Sjåvik, Ole Solheim

**Affiliations:** 1 Department of Neuromedicine and Movement Science, NTNU, Trondheim, Norway; 2 Department of Neurosurgery, Sahlgrenska University Hospital, Blå Stråket 5, vån 3, 41345 Gothenburg, Sweden; 3 Department of Clinical Neuroscience, Institute of Neuroscience and Physiology, University of Gothenburg, Sahlgrenska Academy, Box 430, 40530 Gothenburg, Sweden; 4 Department of Health Research, SINTEF Digital, Trondheim, Norway; 5 Department of Pathology, St. Olavs University Hospital, Trondheim, Norway; 6 Department of Clinical and Molecular Medicine, Faculty of Medicine and Health Sciences, NTNU, Norwegian University of Science and Technology, 7491 Trondheim, Norway; 7 Department of Public Health and Nursing, Faculty of Medicine and Health Sciences, NTNU, Trondheim, Norway; 8 Department of Neurosurgery, St. Olavs University Hospital, Trondheim, Norway; 9 Department of Diagnostic Imaging, Nordland Hospital Trust, Bodø, Norway; 10 Department of Neurosurgery, University Hospital of North Norway, Tromsö, Norway

**Keywords:** Brain neoplasm, Glioma, Neurosurgery, Transformation, Treatment

## Abstract

**Background:**

Malignant transformation represents the natural evolution of diffuse low-grade gliomas (LGG). This is a catastrophic event, causing neurocognitive symptoms, intensified treatment and premature death. However, little is known concerning the spatial distribution of malignant transformation in patients with LGG.

**Materials and methods:**

Patients histopathological diagnosed with LGG and subsequent radiological malignant transformation were identified from two different institutions. We evaluated the spatial distribution of malignant transformation with (1) visual inspection and (2) segmentations of longitudinal tumor volumes. In (1) a radiological transformation site < 2 cm from the tumor on preceding MRI was defined local transformation. In (2) overlap with pretreatment volume after importation into a common space was defined as local transformation. With a centroid model we explored if there were particular patterns of transformations within relevant subgroups.

**Results:**

We included 43 patients in the clinical evaluation, and 36 patients had MRIs scans available for longitudinal segmentations. Prior to malignant transformation, residual radiological tumor volumes were > 10 ml in 93% of patients. The transformation site was considered local in 91% of patients by clinical assessment. Patients treated with radiotherapy prior to transformation had somewhat lower rate of local transformations (83%). Based upon the segmentations, the transformation was local in 92%. We did not observe any particular pattern of transformations in examined molecular subgroups.

**Conclusion:**

Malignant transformation occurs locally and within the T2w hyperintensities in most patients. Although LGG is an infiltrating disease, this data conceptually strengthens the role of loco-regional treatments in patients with LGG.

## Introduction

Diffuse low-grade gliomas WHO grade 2 (LGG) remain a challenging entity in neuro-oncology. These are slow-growing tumors, with a median growth rate of approximately 4 mm/year [1, 2]. In spite of often rather well-defined margins on MRI, tumor cells are present outside the radiologically visible tumor, making this an infiltrative disease [3, 4]. At an unpredictable point of time, LGG speed of growth increases due to malignant transformation [5, 6]. Malignant transformation is a key clinical event and leads to intensified treatment, increased morbidity and premature death [7–9]. Consequently, successful effort to delay transformation is expected to significantly prolong life and preserve quality of life [10].

Compared to studies on recurrence in high-grade gliomas, [11–18] there are relatively few studies concerning patterns of transformation in LGG [19]. The in vivo growth in high-grade gliomas as depicted by MRI tends to follow white matter tracts and spread is less likely to be perpendicular to white matter tracts [20]. Thus, it is also very likely that the malignant transformation in LGG is not random. Drawbacks of the LGG literature concerning transformations are that some papers are old (i.e. from the CT era), mix adults and children, mix grade I and II tumors, or do not clearly differentiate progression from transformation (i.e. “treatment failure”) [21–24]. Nevertheless, a study of 11 malignant transformations, where radiotherapy with 1–3 cm margins was provided, demonstrated that malignant transformation occurred within the irradiated volume [23]. This finding was repeated by two other small studies including 16 and 20 patients with “treatment failures” [22, 24]. A recent review on the topic of progression in LGG pointed out that there are few studies, but following more aggressive therapy there is an impression that atypical and non-local progressions and recurrences more often are seen [19].

Detailed knowledge of patterns of malignant transformation can be useful when providing local and regional treatment. Question remain whether extensive or even supratotal surgical resection is a scientifically sound approach. For instance, supratotal resections does not make much sense if we remove brain unlikely to undergo transformation. Concerning radiotherapy, better knowledge of disease progression can help draw “biological” radiation fields or even provide a scientific ground whether a shift from photon to proton beam radiotherapy is justified. Finally, do the different molecular subgroups have different patterns of transformation? With this background we wanted to study the radiological progression and transformation pattern in more detail.

The aim of this study was to provide detailed data concerning the radiological pattern of malignant transformation in LGG.

## Methods

In this retrospective study, adult patients (18 years or older) with histopathological verified hemispheric diffuse LGG without any contrast enhancement on MRI at time of diagnosis were eligible for inclusion. The patients were recruited from two different institutions, with patients from University Hospital of North Norway included from 1999 through 2009, and at St. Olavs University Hospital from 1999 through 2015. The surgical indications differed between institutions, but the follow-up regimens were similar, but not identical, as described elsewhere [25]. Some patients lacked preoperative images, but in the radiological report it was clearly stated in all cases there was no contrast enhancement. Since the clinical judgement was based upon the pre-transformation scan (see below), we included these patients for the clinical interpretation. The earlier WHO classifications used in the clinical setting were updated to WHO 2016, as previously reported, in all patients with tissue available [8, 26]. In some patients were treated with radio- or chemotherapy before malignant transformation, but in no cases the reason for treatment was new contrast enhancement.

### Malignant transformation

A radiological transformation was considered in the event of a new significant contrast enhancement where repeated scans, clinical course or histopathology from reoperation separated this from cases of pseudoprogression, radionecrosis or unspecific post-treatment changes due to for instance ischemia. To determine the spatial transformation, we relied on MRI findings. Thus, malignant histology from re-operation in the absence of enhancement was despite the transformation excluded from analyses of the spatial distribution of transformation since we had no reliable data on biopsy location. Also, other measures that could be taken as signs of malignant transformation prior to contrast enhancement, such as for instance FET-PET, was not used in this study [27]. Such information would presumably affect the timing of malignant transformation, but we believe to a lesser degree influence the spatial information.

### Spatial distributions

In terms of spatial distribution, we used one method with clinical judgement (A.S.J) with visual inspection and crude one-dimensional measures, and one method based on tumor volume segmentations. A radiological transformation site < 2 cm from the tumor on high-intensity T2w and/or FLAIR signal abnormalities on the pre-transformation MRI scan defined *local* transformation. *Distant* malignant transformation was used if clearly separated (> 2 cm) from the high-signal abnormalities from the pre-transformation scan.

In the method with tumor volume segmentation, semi-automatic segmentations were performed using the open source medical imaging platform 3D Slicer (version 4.8.1, www.slicer.org). A radiologist (H.K.B) performed preoperative segmentations and the follow-up segmentations were performed by a neurosurgeon with extensive experience in radiological LGG assessment (A.S.J). First, we segmented the preoperative tumor volume using T2 or FLAIR MRI sequences. Next, we identified the scan where malignant transformation (i.e. new contrast enhancement) was detected and segmented the contrast enhancement using T1 with gadolinium enhancement. Then, we segmented the T2 or FLAIR volume from the scan prior to the scan where malignant transformation was detected. Together, these segmentations built the fundament to the processing pipeline as described below.

### Processing pipeline

**Fig. 1:**
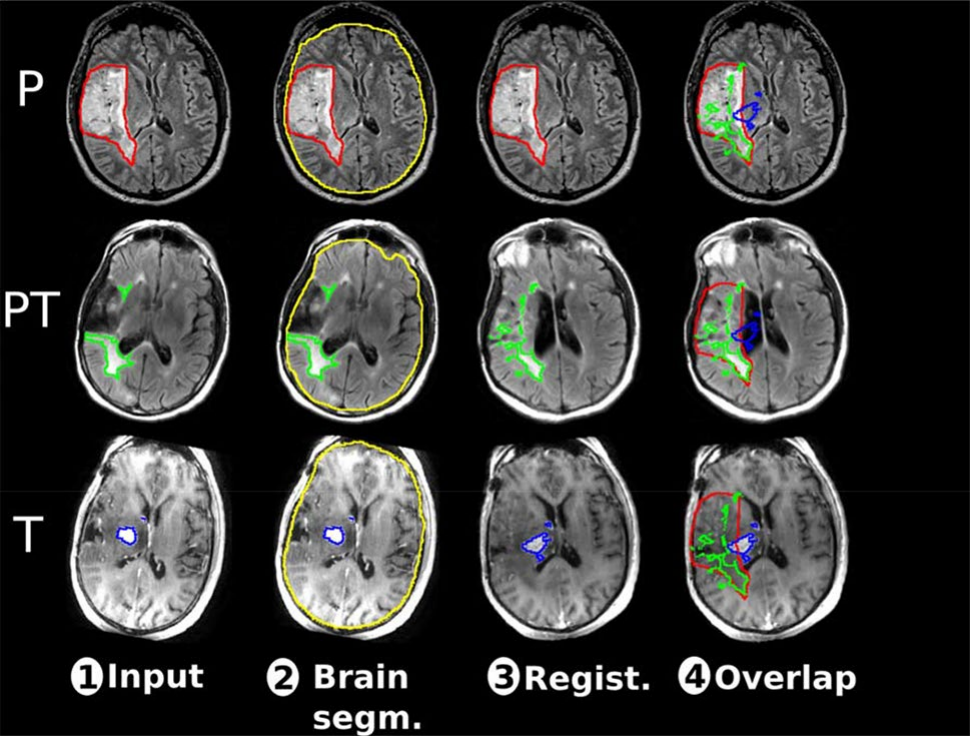
Overview of the processing pipeline to visualize all MRI volumes for a same patient in the same space. The first row (P) represents the pre-operative MRI volume, the second row (PT) the pre-transformation MRI volume, and the third row (T) the transformation MRI volume. The columns are describing for one patient: (1) the initial MRI volumes with manual tumor segmentation (red for pre-operative, green for pre-transformation and blue for transformation), (2) the automatic brain segmentation (in yellow) for skull stripping, (3) the results of the volume registration in the pre-operative space, (4) the post-registration tumor volumes overlap

Key MRI scans being the pre-operative, pre-transformation, and transformation timepoints were selected, and to determine the relative locations of transformation sites they needed to appear in the same referential space. A processing pipeline, illustrated in Fig. 1, was therefore developed to generate the results for each patient. As input, the pair of original images (i.e., pre-operative, pre-transformation, and transformation) and corresponding ground truth volumes (original tumor, pre-transformation, transformation) were used, shown as step 1 in Fig. 1. Then, the brain was automatically segmented using a neural network model pre-trained with over 300 samples, shown as step 2 in Fig. 1. The neural network follows a U-Net architecture and has been implemented in Python using Keras and TensorFlow [28]. Using the brain segmentation, the skull was stripped before performing registration of the pre-transformation and transformation images (as long as the corresponding ground truth volumes) toward the pre-operative MRI volume. This process, illustrated as step 3 in Fig. 1, was performed using a symmetric diffeomorphic technique (named SyN) from the Advanced Normalization Tools [29]. In the end, the three volumes of interest were displayed in an overlap fashion over each MRI volumes, all expressed in the pre-operative MRI space, represented by step 4 in Fig. 1.

### Statistics

In this study we provide only descriptive statistics. This was chosen since focus was to describe patterns of transformation.

## Results

We included 43 patients with radiological transformation in this study and they are presented in Table 1 for an overview. All patients were used in the clinical interpretation, and we had 36 patients with available tumor segmentations where the preoperative volume served as a fundament (seven patients lacked preoperative digitalized MRI images and one patient had a pre-transformation MRI scan that was not possible to segment).

**Table 1:** Baseline, tumor and treatment characteristics for all included patients (n = 43)

Age, mean (SD)	45.2 (12.9)
Female, n (%)	18 (41)
Preoperative tumor volume in ml, median (Q1–Q3)	30 (9–61)*
Histopathology, n (%)	
Oligodendroglioma	12 (28)
Astrocytoma *IDH* mut	16 (37)
Astrocytoma *IDH* wt	13 (30)
LGG, NOS	2 (5)
Surgical resection prior to transformation, n (%)	21 (49)
Chemotherapy prior to transformation, n (%)	14 (33)
Radiotherapy prior to transformation, n (%)	23 (54)

*n = 36 due to lack of digital preoperative MRI

**Fig. 2:**
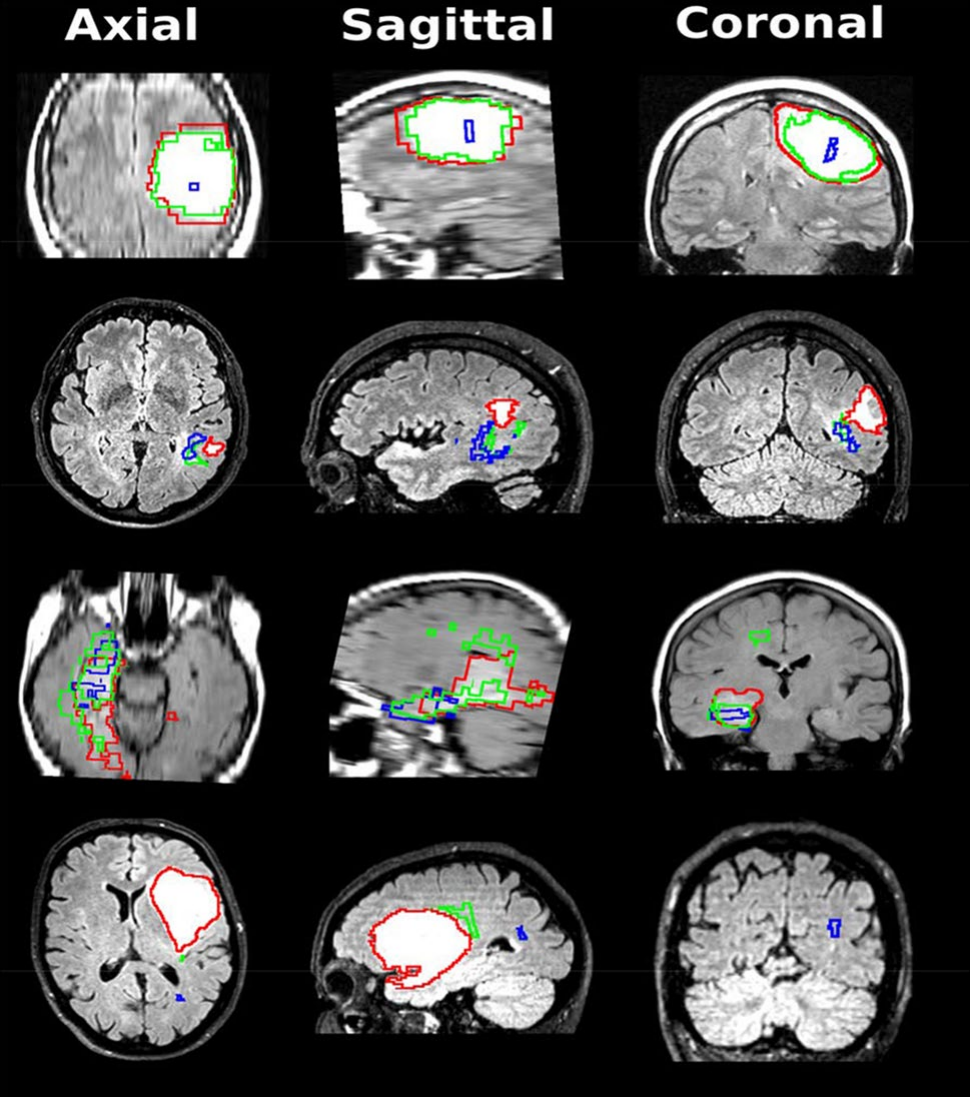
Different examples showcasing the pre-operative volume (in red), the pre-transformation volume (in green) and the transformation volume (in blue) on top of the pre-operative MRI volume (n = 35). Each row is representing a different patient, and each column is representing a different view. First row; local transformation within preoperative volume. Second row; local transformation without overlap. Third row; local transformation with border overlap. Fourth row; distant transformation

In 35/43 patients (81%) we had complete radiological history with preoperative scan, scan prior to transformation (or similar to preoperative scan if rapid transformation), and scan containing the transformation. We used this information to visualize the progression and transformation, and we present a collage of patients with different types of tumor distributions in Fig. 2.

In Tables 2 and 3 we present characteristics relevant to the malignant transformation itself. We observed that 39 patients (91%) had local recurrence according to clinical interpretation. According to the segmentation overlay model, 33/36 patients (92%) had malignant transformation within the preoperative tumor T2w/FLAIR volume. These patients were all considered to have a local transformation based upon clinical judgement, however the one patient with combined distant and local malignant transformation in the clinical data was categorized as local transformation in the overlay model since there was an overlap of volumes.

**Table 2:** Characteristics of malignant transformation (MT) (n = 43)

Median volume of tumor in the pre-transformation MRI, ml (Q1–Q3)	40 (19–89)*
Tumor volume < 10 ml in pre-transformation MRI, n (%)	3/42 (7)*
Median months from pre-transformation MRI scan to MT (Q1–Q3)	5 (3–12)
Multifocal MT, n (%)	11 (26)
MT volume in ml, median (Q1–Q3)	1.4 (0.4–5.0)
Median months from first surgery until MT (Q1–Q3)	37 (13–70)
Clinical; local MT, n (%)	
Local (within 2 cm), n (%)	39 (91)
Distant, n (%)	3 (7)
Combined, n (%)	1 (2)
Model; MT within preoperative volume, n (%)	33/36 (92)**
Histopathological verified MT through new resection, n (%)	19 (44)

*n = 42 due to volumetric analysis not possible in one of the pre-transformation scans

**n = 36 due to lack of digital preoperative MRI

**Table 3:** Patterns of malignant transformation (MT) according to clinical judgement and according to the model using tumor volumes overlay for clinically relevant subgroups

	Clinic: local MT	Model: MT inside pre-op
Astrocytoma *IDH* wt, n/N (%)	13/13 (100)	13/13 (100)
Astrocytoma *IDH* mut, n/N (%)	14/16 (88)	10/11 (91)
Oligodendroglioma, n/N (%)	11/12 (92)	9/10 (90)
Chemotherapy prior to MT, n/N (%)	13/14 (93)	12/13 (92)
Radiotherapy prior to MT, n/N (%)	19/23 (83)	15/18 (83)
No resection prior to MT, n/N (%)	20/22 (91)	20/22 (91)
Resection prior to MT, n/N (%)	19/21 (91)	13/14 (93)

When we used a model of malignant transformation with pre-transformation images instead of pre-operative images, there were no relevant differences in results (n = 43)

*MT denotes malignant transformation

**Fig. 3:**
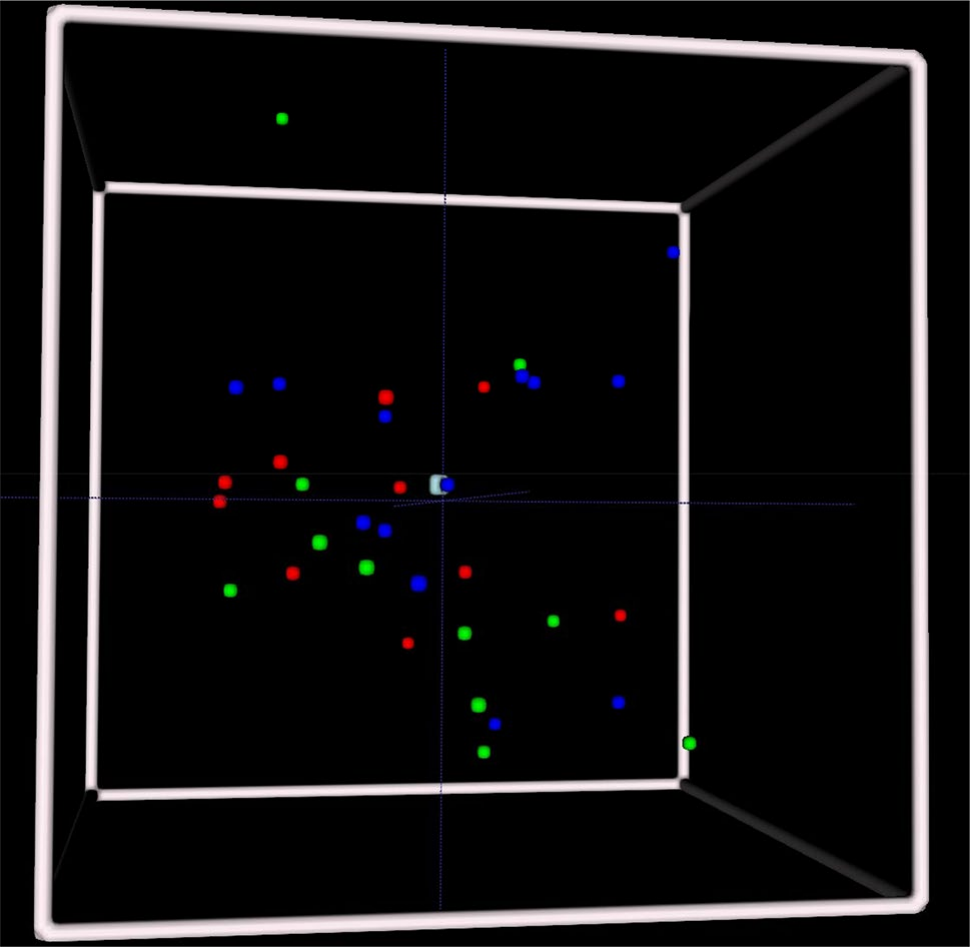
Overall distribution of the relative position of the centroid of the transformation volume over the centroid of the pre-operative volume for the different molecular marker groups. The few distant transformations are excluded in this model (n = 3). White represents the borders of the unitary pre-operative volume, red represents oligodendroglioma, green the *IDH* mut astrocytomas, and blue the *IDH* wt astrocytomas

An overall representation of the relative location of the transformation inside the pre-operative tumoral volume is shown in Fig. 3. A simplified tumor volume is represented as a unitary cube, where the center of cube reflects the center of the tumor. The distance ratio between the centroid of the transformation volume (after registration) and the centroid of the preoperative tumor volume is computed and is represented as one colored dot in the figure. A central dot means a transformation happened in the middle of the pre-operative tumor, and a dot closer to the white edges means a transformation happening on the border of the pre-operative tumor. Overall, there were many transformations occurring in a central location with respect to the preoperative tumor volume. In Fig. 3, we also visualize transformation sites for the different molecular subgroups, but no obvious pattern was seen.

We further summarized the pattern of transformation (according to clinical judgement, n = 43) in molecular subgroups and in relation to therapy given. There was no obvious pattern in local versus distant transformation, but the group with radiotherapy had somewhat lower proportion of local transformation (83%).

## Discussion

This study shows that malignant transformation of LGG most often occurs locally regardless of subgroups. We found no specific transformation patterns with respect to molecular subgroups or when separated by type of treatment. Although LGG is an infiltrative cancer and MRI is insensitive in terms of detecting the biological tumor volume, almost all cases of transformation do occur locally within or in close proximity to previous areas of hyperintensities as seen in T2w sequences.

### Tumor burden

Studies indicate an association between tumor size or size of tumor remnant and earlier malignant transformation [7, 30–34]. The dose–response relationship of tumor size and malignant transformation in astrocytomas and oligoastrocytomas was demonstrated by Shaw et al. where 28% recurred with < 1 cm of remnant, 88% where remnant was 1–2 cm and 100% if > 2 cm remnant [33]. For oligodendrogliomas, the dose response relationship was also present, but to lesser extent with 23%, 43% and 75% depending on largest diameter of remnant. Of note, in the study from Shaw et al. the definition of progression was a clear increase in T2/FLAIR or contrast enhancement, and this differs from our definition focusing on the detrimental event of malignant transformation. Others have demonstrated that oligodendrogliomas are more likely to progress without malignant transformation [30, 35]. Since the existing literature is a mix of progression and transformation, the numbers would likely differ even more between astrocytomas and oligodendrogliomas if only malignant transformation was analyzed.

In the surgical literature several studies have reported that remnant less than 10–15 ml have a better prognosis [36, 37] although no visible postoperative remnant is clearly superior [38–40]. In our series, smaller residual tumor volumes than 10 ml did not protect against transformation although it was seen in only 7% of cases. Also, in none of the patients transformation occurred without any preceding T2 hyperintensities, albeit in almost 10% of cases the transformations were distant and seemingly unrelated to earlier T2w hyperintensities. This association with MRI defined volume and transformation is further corroborated by a study that focused on recurrent surgery of a previous LGG, where in stable lesions being WHO grade II the median volume was 15.6 ml while for tumors with transformations to WHO grade III and grade IV the volumes were 30.9 ml and 69.7 ml volume respectively [34].

### Spatial distribution

In perceived low-risk patients undergoing surgical resection with aim of gross-total removal, and where no adjuvant therapy was provided, the spatial distribution of progression was found to be within 2 cm from the resection cavity in 82% of cases, more than 2 cm away in 16% and 2% had truly distant progression [33]. In our cohort that was not a typical low-risk profile and being based partly upon historical data where biopsies where frequently performed [25], we observed a higher proportion of local progressions and transformations. In low-risk patients undergoing extensive resection the pattern of transformation may be somewhat different, as observed by others that more aggressive therapy leave room for more atypical and distant progression and transformation patterns in the longer-term [19]. We included only patients with radiological transformations, excluding patients with transformation based upon histopathology alone from reoperation without preceding change in MR phenotype. These surgical transformations are also local in origin, hence the vast majority of patients with LGG will present a local transformation. Overall, the patterns of distributions together with the volume associations mentioned above *conceptually* favor aggressive locoregional therapy, and presumably also repeated surgery whenever possible.

An early study from North et al. demonstrated that all treatment failures were within the radiation field when 2–3 cm margin was used [21]. Similarly, Shaw et al. described that all failures were within the radiation field [24]. Also, survival was not improved by more extensive fields such as whole brain radiotherapy [24]. We observed slightly more distant recurrences in the subgroup treated with radiotherapy prior to malignant transformation. This may be a survivor effect if radiotherapy to some degree prevents local malignant transformation, and since there is no cure, transformations will therefore eventually more often be distant. A recent study in patients with anaplastic gliomas treated with intensity-modulated radiation therapy suggested that radiotherapy may prevent local recurrence. In that study, a relapse pattern with components of marginal and distant pattern were observed in 19% and 37%, respectively [41]. In our view, the majority of studies indicate that the majority of progression and transformation follow a local pattern, and this can argue for proton-beam radiotherapy in patients with LGG. However, to date there is very limited clinical evidence [42]. It has been speculated if the more conformal field would create a risk for more distant recurrences. However, one very recent larger retrospective study found that most recurrences following proton beam-radiotherapy were indeed local, with only 12% being “out of field”, a comparable figure to our “distant” transformations [43]. Thus, the “dose-bath” beyond the targeted areas are perhaps not needed since most recurrences and transformations are local. That larger areas of the presumably functional brain more often receives no radiation based upon comparative proton plans, means also less risk in the longer-term for cognitive decline. This is potentially important as we otherwise can transform long-term treatment successes in terms of survival to long-term failures speaking of cognitive function and quality of life.

### Molecular markers and patterns of radiological transformation

Previous studies of malignant transformation have not used the WHO 2016 classification, and this may affect results. One recent study on failures following radiotherapy in anaplastic gliomas demonstrated a distant pattern of failure in 45% of *IDH* mutated patients compared to 25% in those with *IDH*wt [41]. In our study we had > 1/3 with *IDH* mutated astrocytomas and almost 30% molecularly defined oligodendrogliomas, however we did not find any differences in patterns of recurrence with respect to the molecular profile of the tumors. However, across subgroups the most common pattern of transformation was local.

### Limitations

Ideally all areas should have been sampled to verify malignant transformation, although either new histopathology or the clinical course ensured that only patients with malignant transformation were included (and not pseudoprogression). The T2/FLAIR volume at time of progression was segmented to illustrate the growth of the tumor and use this in relation to the newly developed contrast enhancement to demonstrate the tumor evolution. However, in some cases the T2w images showed the occurrence of gliosis following surgery and hyperintensities in patients undergoing radiotherapy. In these cases, this volume is associated with inherent uncertainty. Also, even the intra-observer variability in LGG segmentations can be significant [44]. The overlay segmentation model also holds some limitations when comparing with the preoperative volumes in patients undergoing resection, as the cavity may shrink/collapse causing areas outside the cavity to appear within the cavity perhaps increasing the proportion of recurrences within the preoperative tumor volume. Further, this study is not equipped to answer effectiveness of therapies and the sample size did not allow for comparisons for time to transformation. Also, the small sample limits the subgroup analyses. Finally, the centroid model can provide erroneous results in multifocal tumors.

## Conclusion

We provide new data on malignant transformation in patients with LGG. Although the tumors are diffusely infiltrating brain tissue outside the radiological tumor, the catastrophic event of malignant transformation occurs locally in the vast majority of patients. Molecular subgroups exhibit the same patterns of transformation. Minimizing the dense tumor, as defined by hyperintensity in T2w images, may prolong time to transformation. This may explain the strong association of extensive resections and survival, and conceptually strengthen the role of effective loco-regional treatments in patients with LGG.

## Data Availability

All data generated or analyzed during this study are included in this published article.
